# {1,1′-[Butane-1,4-diylbis(nitrilo­methyl­idyne)]di-2-naphtho­lato}copper(II) ethanol monosolvate

**DOI:** 10.1107/S1600536810053183

**Published:** 2010-12-24

**Authors:** Hadi Kargar, Reza Kia

**Affiliations:** a Department of Chemistry, School of Science, Payame Noor University (PNU), Ardakan, Yazd, Iran; b Department of Chemistry, Science and Research Branch, Islamic Azad University, Tehran, Iran; c X-ray Crystallography Laboratory, Plasma Physics Research Center, Science and Research Branch, Islamic Azad University, Tehran, Iran

## Abstract

The asymmetric unit of the title compound, [Cu(C_26_H_22_N_2_O_2_)]·C_2_H_5_OH, comprises a Schiff base complex and an ethanol mol­ecule of crystallization. The Cu^II^ atom shows a distorted square-planar geometry. The dihedral angle between the two aromatic rings is 48.16 (13)°. The crystal structure is stabilized by inter­molecular O—H⋯O and C—H⋯O hydrogen bonds and inter­molecular π–π inter­actions with centroid–centroid distances in the range 3.485 (2)–3.845 (3) Å.

## Related literature

For standard values of bond lengths, see: Allen *et al.* (1987 ▶). For background to Schiff base–metal complexes, see: Granovski *et al.* (1993 ▶); Blower *et al.* (1998 ▶); Elmali *et al.* (2000 ▶); Kargar *et al.* (2010 ▶).
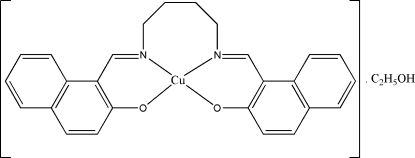


## Experimental

### 

#### Crystal data

[Cu(C_26_H_22_N_2_O_2_)]·C_2_H_6_O
*M* *_r_* = 504.06
Monoclinic, 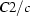

*a* = 13.468 (3) Å
*b* = 22.606 (5) Å
*c* = 15.831 (3) Å
β = 95.84 (3)°
*V* = 4794.9 (17) Å^3^
*Z* = 8
Mo *K*α radiation
μ = 0.94 mm^−1^
*T* = 296 K
0.42 × 0.26 × 0.22 mm

#### Data collection

Stoe IPDS II image plate diffractometer
Absorption correction: multi-scan (*MULABS* in *PLATON*; Spek, 2009 ▶) *T*_min_ = 0.973, *T*_max_ = 1.000
9868 measured reflections
4655 independent reflections
3110 reflections with *I* > 2σ(*I*)
*R*_int_ = 0.053

#### Refinement

*R*[*F* ^2^ > 2σ(*F* ^2^)] = 0.057
*wR*(*F* ^2^) = 0.126
*S* = 1.04
4655 reflections
308 parameters
H-atom parameters constrained
Δρ_max_ = 0.55 e Å^−3^
Δρ_min_ = −0.29 e Å^−3^

### 

Data collection: *X-AREA* (Stoe & Cie, 2005 ▶); cell refinement: *X-AREA*; data reduction: *X-RED32* (Stoe & Cie, 2005 ▶); program(s) used to solve structure: *SHELXTL* (Sheldrick, 2008 ▶); program(s) used to refine structure: *SHELXTL*; molecular graphics: *SHELXTL*; software used to prepare material for publication: *SHELXTL* and *PLATON* (Spek, 2009 ▶).

## Supplementary Material

Crystal structure: contains datablocks global, I. DOI: 10.1107/S1600536810053183/jh2247sup1.cif

Structure factors: contains datablocks I. DOI: 10.1107/S1600536810053183/jh2247Isup2.hkl

Additional supplementary materials:  crystallographic information; 3D view; checkCIF report

## Figures and Tables

**Table 1:** Hydrogen-bond geometry (Å, °)

*D*—H⋯*A*	*D*—H	H⋯*A*	*D*⋯*A*	*D*—H⋯*A*
O3—H1⋯O1	0.90	1.95	2.837 (4)	167
C12—H12*A*⋯O2^i^	0.97	2.52	3.395 (5)	150

Symmetry code: (i) 
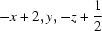
.

**Fig. 1.**
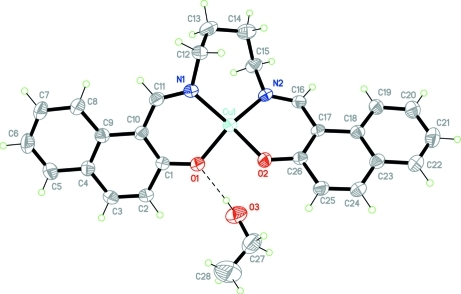
The asymmetric unit of the title compound, showing 40% probability displacement ellipsoids and the atomic numbering. The intermolecular interaction is shown as dashed lines.

**Fig. 2.**
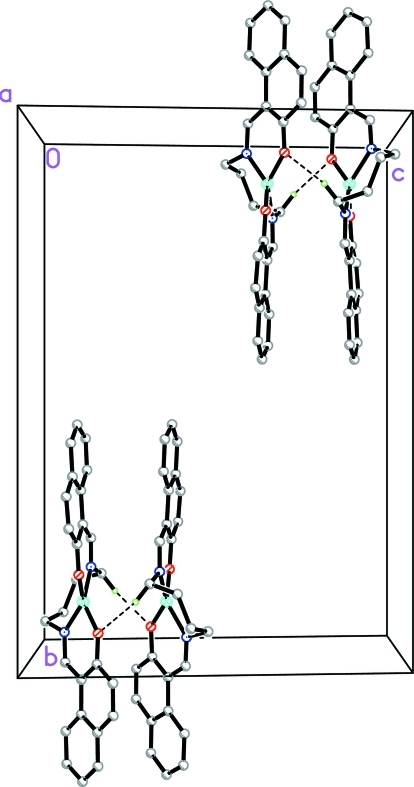
The packing of the title compound viewed down the a-axis showing dimer formation through the intermolecular C—H···O hydrogen bonds. All H atoms were removed for clarity except those involved in the hydrogen bonding. The intermolecular interactions are shown as dashed lines.

## supplementary crystallographic information

### Comment

Schiff base complexes are one of the most important stereochemical models in transition metal coordination chemistry, with the ease of preparation and structural variations (Granovski et al., 1993). Metal derivatives of the Schiff bases have been studied extensively, and Ni(II) and Cu(II) complexes play a major role in both synthetic and structurel research (Kargar et al., 2010; Elmali et al., 2000; Blower et al., 1998).

The asymmetric unit of the title compound, Fig. 1, comprises one unit of the Schiff base complex and an ethanol molecule of crystallization. The bond lengths (Allen et al., 1987) and angles are within the normal ranges. The geometry ground the Cu(II) atom is distorted square-planar which is coordinated by the N_2_O_2_ donor atoms of the desired potentially tetradenate Schiff base ligand. The dihedral angle between the two aromatic rings is 48.16 (13)°. The crystal structure is stabilized by the intermolecular O—H···O and C—H···O hydrogen bonds and intermolecular π–π interactions [Cg1···Cg1^i^ = 3.4852 (18) Å, (i) 2 - x, y, 1/2 - z; Cg2···Cg2^i^ = 3.7183 (6)Å; Cg3···Cg3^i^ = 3.638 (2)Å; Cg4···Cg4^i^ = 3.845 (3)Å], Cg1, Cg2, Cg3 and Cg4 are the centroids of the Cu1/O1/C1/C10/C11/N1, Cu1/O2/C26/C17/C16/N2, C17/C18/C23/C24/C25/C26, and C18–C23 rings, respectively.

### Experimental

The title compound was synthesized by adding bis(hydroxy naphtylidene)-1,4-butanediamine (2 mmol) to a solution of CuCl_2_. 4 H_2_O (2 mmol) in ethanol (30 ml). The mixture was refluxed with stirring for half an hour. The resultant green solution was filtered. Dark-green block single crystals of the title compound suitable for *X*-ray structure determination were recrystallized from ethanol by slow evaporation of the solvents at room temperature over several days.

### Refinement

All hydrogen atoms were positioned geometrically with C—H = 0.93-0.97 Å and included in a riding model approximation with U_iso_ (H) = 1.2 or 1.5 U_eq_ (C). The H atom of the hydroxy group was located from the difference Fourier mapand constrained to refine with the parent atom with U_iso_ (H) = 1.5 U_eq_ (O)  after its distance was restrained to 0.90 (1)Å.

### Crystal data

**Table:**

[Cu(C_26_H_22_N_2_O_2_)]·C_2_H_6_O	*F*(000) = 2104
*M**_r_* = 504.06	*D*_x_ = 1.397 Mg m^−^^3^
Monoclinic, *C*2/*c*	Mo *K*α radiation, λ = 0.71073 Å
Hall symbol: -C 2yc	Cell parameters from 10437 reflections
*a* = 13.468 (3) Å	θ = 1.8–29.6°
*b* = 22.606 (5) Å	µ = 0.94 mm^−^^1^
*c* = 15.831 (3) Å	*T* = 296 K
β = 95.84 (3)°	Block, dark-green
*V* = 4794.9 (17) Å^3^	0.42 × 0.26 × 0.22 mm
*Z* = 8	

### Data collection

**Table:**

Stoe IPDS II image plate diffractometer	4655 independent reflections
Radiation source: fine-focus sealed tube	3110 reflections with *I* > 2σ(*I*)
graphite	*R*_int_ = 0.053
Detector resolution: 0.15 mm pixels mm^-1^	θ_max_ = 26.0°, θ_min_ = 1.8°
ω scans	*h* = −16→16
Absorption correction: multi-scan (*MULABS* in *PLATON*; Spek, 2009)	*k* = −27→27
*T*_min_ = 0.973, *T*_max_ = 1.000	*l* = −14→19
9868 measured reflections	

### Refinement

**Table:**

Refinement on *F*^2^	Primary atom site location: structure-invariant direct methods
Least-squares matrix: full	Secondary atom site location: difference Fourier map
*R*[*F*^2^ > 2σ(*F*^2^)] = 0.057	Hydrogen site location: inferred from neighbouring sites
*wR*(*F*^2^) = 0.126	H-atom parameters constrained
*S* = 1.04	*w* = 1/[σ^2^(*F*_o_^2^) + (0.0612*P*)^2^] where *P* = (*F*_o_^2^ + 2*F*_c_^2^)/3
4655 reflections	(Δ/σ)_max_ = 0.001
308 parameters	Δρ_max_ = 0.55 e Å^−^^3^
0 restraints	Δρ_min_ = −0.29 e Å^−^^3^

### Special details

**Table:**

Geometry. All esds (except the esd in the dihedral angle between two l.s. planes) are estimated using the full covariance matrix. The cell esds are taken into account individually in the estimation of esds in distances, angles and torsion angles; correlations between esds in cell parameters are only used when they are defined by crystal symmetry. An approximate (isotropic) treatment of cell esds is used for estimating esds involving l.s. planes.
Refinement. Refinement of F^2^ against ALL reflections. The weighted R-factor wR and goodness of fit S are based on F^2^, conventional R-factors R are based on F, with F set to zero for negative F^2^. The threshold expression of F^2^ > 2sigma(F^2^) is used only for calculating R-factors(gt) etc. and is not relevant to the choice of reflections for refinement. R-factors based on F^2^ are statistically about twice as large as those based on F, and R- factors based on ALL data will be even larger.

### Fractional atomic coordinates and isotropic or equivalent isotropic displacement parameters (Å^2^)

**Table:**

	*x*	*y*	*z*	*U*_iso_*/*U*_eq_	
Cu1	0.99074 (3)	0.39671 (2)	0.13736 (3)	0.03967 (16)	
O1	1.09554 (19)	0.34050 (11)	0.13087 (19)	0.0462 (7)	
O2	1.0912 (2)	0.45001 (11)	0.18187 (19)	0.0475 (7)	
N1	0.8934 (2)	0.33537 (13)	0.1535 (2)	0.0402 (8)	
N2	0.9081 (2)	0.45888 (13)	0.08166 (19)	0.0368 (7)	
C1	1.0894 (3)	0.28282 (16)	0.1258 (2)	0.0370 (9)	
C2	1.1777 (3)	0.25202 (17)	0.1090 (3)	0.0465 (10)	
H2A	1.2354	0.2734	0.1025	0.056*	
C3	1.1793 (3)	0.19265 (17)	0.1022 (3)	0.0475 (10)	
H3A	1.2376	0.1741	0.0895	0.057*	
C4	1.0948 (3)	0.15794 (16)	0.1140 (3)	0.0419 (9)	
C5	1.0986 (3)	0.09535 (18)	0.1096 (3)	0.0535 (11)	
H5A	1.1573	0.0769	0.0977	0.064*	
C6	1.0179 (4)	0.06193 (19)	0.1226 (3)	0.0627 (13)	
H6A	1.0216	0.0209	0.1195	0.075*	
C7	0.9301 (4)	0.08883 (17)	0.1405 (3)	0.0596 (13)	
H7A	0.8752	0.0658	0.1502	0.072*	
C8	0.9236 (3)	0.14926 (17)	0.1440 (3)	0.0506 (11)	
H8A	0.8636	0.1665	0.1549	0.061*	
C9	1.0052 (3)	0.18599 (17)	0.1315 (2)	0.0380 (8)	
C10	1.0024 (3)	0.25057 (16)	0.1367 (3)	0.0372 (8)	
C11	0.9126 (3)	0.27942 (16)	0.1541 (2)	0.0395 (9)	
H11A	0.8612	0.2548	0.1676	0.047*	
C12	0.7936 (3)	0.35263 (18)	0.1772 (3)	0.0470 (10)	
H12A	0.8013	0.3864	0.2151	0.056*	
H12B	0.7670	0.3203	0.2082	0.056*	
C13	0.7198 (3)	0.3680 (2)	0.1035 (3)	0.0630 (13)	
H13A	0.6532	0.3625	0.1204	0.076*	
H13B	0.7278	0.3403	0.0579	0.076*	
C14	0.7270 (3)	0.4312 (2)	0.0682 (3)	0.0595 (12)	
H14A	0.6686	0.4387	0.0285	0.071*	
H14B	0.7256	0.4590	0.1147	0.071*	
C15	0.8183 (3)	0.4431 (2)	0.0241 (3)	0.0486 (10)	
H15A	0.8331	0.4082	−0.0079	0.058*	
H15B	0.8039	0.4751	−0.0160	0.058*	
C16	0.9315 (3)	0.51465 (16)	0.0851 (2)	0.0359 (8)	
H16A	0.8889	0.5400	0.0524	0.043*	
C17	1.0155 (2)	0.54158 (15)	0.1335 (2)	0.0320 (8)	
C18	1.0243 (3)	0.60666 (17)	0.1334 (2)	0.0346 (8)	
C19	0.9549 (3)	0.64393 (17)	0.0881 (3)	0.0437 (10)	
H19A	0.9002	0.6277	0.0557	0.052*	
C20	0.9669 (4)	0.70446 (19)	0.0913 (3)	0.0589 (12)	
H20A	0.9195	0.7286	0.0615	0.071*	
C21	1.0488 (4)	0.7300 (2)	0.1382 (4)	0.0707 (15)	
H21A	1.0564	0.7709	0.1391	0.085*	
C22	1.1174 (3)	0.69509 (19)	0.1827 (3)	0.0584 (12)	
H22A	1.1716	0.7123	0.2148	0.070*	
C23	1.1074 (3)	0.63268 (17)	0.1808 (3)	0.0406 (9)	
C24	1.1801 (3)	0.59615 (17)	0.2255 (3)	0.0441 (9)	
H24A	1.2346	0.6138	0.2566	0.053*	
C25	1.1733 (3)	0.53693 (17)	0.2245 (3)	0.0429 (9)	
H25A	1.2232	0.5144	0.2540	0.052*	
C26	1.0900 (3)	0.50794 (16)	0.1786 (2)	0.0362 (8)	
O3	1.2478 (2)	0.40229 (16)	0.0561 (2)	0.0741 (10)	
H1	1.2027	0.3859	0.0872	0.111*	
C27	1.3277 (4)	0.4255 (3)	0.1075 (4)	0.0863 (18)	
H27A	1.3024	0.4447	0.1557	0.104*	
H27B	1.3594	0.4556	0.0758	0.104*	
C28	1.4013 (6)	0.3832 (4)	0.1383 (5)	0.131 (3)	
H28A	1.4506	0.4021	0.1774	0.197*	
H28B	1.4328	0.3673	0.0915	0.197*	
H28C	1.3702	0.3519	0.1668	0.197*	

### Atomic displacement parameters (Å^2^)

**Table:**

	*U*^11^	*U*^22^	*U*^33^	*U*^12^	*U*^13^	*U*^23^
Cu1	0.0315 (2)	0.0356 (2)	0.0519 (3)	−0.0033 (2)	0.00417 (19)	0.0020 (3)
O1	0.0345 (14)	0.0385 (15)	0.066 (2)	−0.0025 (12)	0.0089 (14)	−0.0030 (13)
O2	0.0387 (15)	0.0332 (14)	0.067 (2)	−0.0018 (12)	−0.0109 (14)	0.0052 (13)
N1	0.0309 (16)	0.0389 (17)	0.052 (2)	−0.0010 (13)	0.0092 (15)	0.0013 (15)
N2	0.0295 (16)	0.0404 (17)	0.0401 (19)	−0.0068 (13)	0.0021 (14)	−0.0015 (14)
C1	0.0316 (18)	0.035 (2)	0.045 (2)	0.0009 (15)	0.0031 (17)	−0.0003 (17)
C2	0.032 (2)	0.044 (2)	0.064 (3)	−0.0017 (16)	0.0079 (19)	0.003 (2)
C3	0.035 (2)	0.045 (2)	0.063 (3)	0.0073 (17)	0.006 (2)	−0.003 (2)
C4	0.043 (2)	0.037 (2)	0.045 (2)	−0.0006 (17)	0.0026 (18)	−0.0003 (17)
C5	0.053 (2)	0.041 (2)	0.066 (3)	0.006 (2)	0.005 (2)	−0.005 (2)
C6	0.075 (3)	0.032 (2)	0.081 (4)	−0.004 (2)	0.011 (3)	−0.005 (2)
C7	0.059 (3)	0.034 (2)	0.087 (4)	−0.0127 (19)	0.011 (3)	−0.009 (2)
C8	0.047 (2)	0.040 (2)	0.066 (3)	−0.0065 (19)	0.008 (2)	−0.002 (2)
C9	0.038 (2)	0.0394 (19)	0.037 (2)	−0.0013 (17)	0.0034 (17)	−0.0035 (18)
C10	0.035 (2)	0.0358 (18)	0.041 (2)	−0.0052 (16)	0.0022 (17)	−0.0029 (18)
C11	0.0342 (19)	0.041 (2)	0.044 (2)	−0.0055 (16)	0.0071 (17)	0.0002 (17)
C12	0.038 (2)	0.045 (2)	0.061 (3)	0.0015 (18)	0.017 (2)	0.000 (2)
C13	0.038 (2)	0.065 (3)	0.086 (4)	−0.013 (2)	0.002 (2)	0.007 (3)
C14	0.042 (2)	0.072 (3)	0.062 (3)	−0.002 (2)	−0.006 (2)	−0.001 (2)
C15	0.039 (2)	0.058 (3)	0.045 (3)	−0.0073 (19)	−0.0100 (19)	−0.001 (2)
C16	0.0298 (19)	0.040 (2)	0.038 (2)	0.0009 (16)	0.0029 (17)	0.0031 (16)
C17	0.0258 (18)	0.0373 (19)	0.033 (2)	−0.0036 (14)	0.0047 (16)	0.0020 (16)
C18	0.0326 (16)	0.0403 (19)	0.0318 (19)	−0.0016 (17)	0.0084 (15)	−0.0026 (18)
C19	0.043 (2)	0.039 (2)	0.048 (3)	0.0026 (18)	0.000 (2)	0.0033 (18)
C20	0.057 (3)	0.042 (2)	0.076 (3)	0.003 (2)	0.000 (3)	0.009 (2)
C21	0.074 (3)	0.038 (2)	0.099 (4)	−0.007 (2)	0.001 (3)	0.003 (3)
C22	0.048 (3)	0.046 (2)	0.079 (4)	−0.009 (2)	0.000 (2)	−0.007 (2)
C23	0.039 (2)	0.043 (2)	0.041 (2)	−0.0053 (17)	0.0078 (18)	−0.0022 (18)
C24	0.038 (2)	0.046 (2)	0.047 (2)	−0.0073 (18)	−0.0022 (17)	−0.0042 (19)
C25	0.035 (2)	0.044 (2)	0.049 (3)	−0.0023 (17)	−0.0026 (18)	0.0040 (18)
C26	0.0293 (18)	0.038 (2)	0.042 (2)	−0.0029 (15)	0.0075 (17)	−0.0005 (17)
O3	0.0546 (19)	0.092 (3)	0.077 (2)	−0.013 (2)	0.0114 (17)	0.008 (2)
C27	0.075 (4)	0.075 (4)	0.110 (5)	−0.018 (3)	0.012 (4)	−0.020 (3)
C28	0.099 (5)	0.142 (7)	0.143 (8)	0.010 (5)	−0.033 (5)	0.022 (6)

### Geometric parameters (Å, °)

**Table:**

Cu1—O2	1.893 (3)	C13—H13B	0.9700
Cu1—O1	1.910 (3)	C14—C15	1.498 (6)
Cu1—N1	1.943 (3)	C14—H14A	0.9700
Cu1—N2	1.947 (3)	C14—H14B	0.9700
O1—C1	1.308 (4)	C15—H15A	0.9700
O2—C26	1.311 (4)	C15—H15B	0.9700
N1—C11	1.291 (5)	C16—C17	1.436 (5)
N1—C12	1.485 (4)	C16—H16A	0.9300
N2—C16	1.299 (4)	C17—C26	1.397 (5)
N2—C15	1.481 (5)	C17—C18	1.476 (5)
C1—C10	1.405 (5)	C18—C19	1.400 (5)
C1—C2	1.427 (5)	C18—C23	1.411 (5)
C2—C3	1.347 (5)	C19—C20	1.378 (6)
C2—H2A	0.9300	C19—H19A	0.9300
C3—C4	1.411 (5)	C20—C21	1.391 (7)
C3—H3A	0.9300	C20—H20A	0.9300
C4—C9	1.415 (5)	C21—C22	1.357 (7)
C4—C5	1.418 (5)	C21—H21A	0.9300
C5—C6	1.356 (6)	C22—C23	1.417 (6)
C5—H5A	0.9300	C22—H22A	0.9300
C6—C7	1.384 (6)	C23—C24	1.414 (5)
C6—H6A	0.9300	C24—C25	1.342 (5)
C7—C8	1.370 (6)	C24—H24A	0.9300
C7—H7A	0.9300	C25—C26	1.431 (5)
C8—C9	1.406 (5)	C25—H25A	0.9300
C8—H8A	0.9300	O3—C27	1.384 (6)
C9—C10	1.463 (5)	O3—H1	0.9000
C10—C11	1.426 (5)	C27—C28	1.427 (8)
C11—H11A	0.9300	C27—H27A	0.9700
C12—C13	1.495 (6)	C27—H27B	0.9700
C12—H12A	0.9700	C28—H28A	0.9600
C12—H12B	0.9700	C28—H28B	0.9600
C13—C14	1.541 (6)	C28—H28C	0.9600
C13—H13A	0.9700		
			
O2—Cu1—O1	86.54 (12)	C15—C14—C13	114.9 (4)
O2—Cu1—N1	150.64 (14)	C15—C14—H14A	108.6
O1—Cu1—N1	92.55 (12)	C13—C14—H14A	108.6
O2—Cu1—N2	93.61 (12)	C15—C14—H14B	108.6
O1—Cu1—N2	147.92 (13)	C13—C14—H14B	108.6
N1—Cu1—N2	102.26 (13)	H14A—C14—H14B	107.5
C1—O1—Cu1	128.6 (2)	N2—C15—C14	114.5 (4)
C26—O2—Cu1	128.0 (3)	N2—C15—H15A	108.6
C11—N1—C12	116.2 (3)	C14—C15—H15A	108.6
C11—N1—Cu1	124.3 (3)	N2—C15—H15B	108.6
C12—N1—Cu1	119.1 (2)	C14—C15—H15B	108.6
C16—N2—C15	116.0 (3)	H15A—C15—H15B	107.6
C16—N2—Cu1	123.8 (3)	N2—C16—C17	127.4 (4)
C15—N2—Cu1	119.8 (2)	N2—C16—H16A	116.3
O1—C1—C10	124.0 (3)	C17—C16—H16A	116.3
O1—C1—C2	116.7 (3)	C26—C17—C16	121.9 (3)
C10—C1—C2	119.4 (3)	C26—C17—C18	119.3 (3)
C3—C2—C1	121.4 (4)	C16—C17—C18	118.8 (3)
C3—C2—H2A	119.3	C19—C18—C23	118.2 (4)
C1—C2—H2A	119.3	C19—C18—C17	123.4 (3)
C2—C3—C4	121.6 (4)	C23—C18—C17	118.4 (3)
C2—C3—H3A	119.2	C20—C19—C18	120.6 (4)
C4—C3—H3A	119.2	C20—C19—H19A	119.7
C3—C4—C9	119.5 (3)	C18—C19—H19A	119.7
C3—C4—C5	121.0 (4)	C19—C20—C21	121.0 (5)
C9—C4—C5	119.5 (4)	C19—C20—H20A	119.5
C6—C5—C4	121.0 (4)	C21—C20—H20A	119.5
C6—C5—H5A	119.5	C22—C21—C20	119.8 (4)
C4—C5—H5A	119.5	C22—C21—H21A	120.1
C5—C6—C7	120.0 (4)	C20—C21—H21A	120.1
C5—C6—H6A	120.0	C21—C22—C23	120.6 (4)
C7—C6—H6A	120.0	C21—C22—H22A	119.7
C8—C7—C6	120.4 (4)	C23—C22—H22A	119.7
C8—C7—H7A	119.8	C18—C23—C24	119.6 (3)
C6—C7—H7A	119.8	C18—C23—C22	119.7 (4)
C7—C8—C9	121.9 (4)	C24—C23—C22	120.7 (4)
C7—C8—H8A	119.0	C25—C24—C23	122.2 (4)
C9—C8—H8A	119.0	C25—C24—H24A	118.9
C8—C9—C4	117.1 (4)	C23—C24—H24A	118.9
C8—C9—C10	123.9 (4)	C24—C25—C26	120.8 (4)
C4—C9—C10	119.0 (3)	C24—C25—H25A	119.6
C1—C10—C11	121.4 (3)	C26—C25—H25A	119.6
C1—C10—C9	119.0 (3)	O2—C26—C17	124.7 (3)
C11—C10—C9	119.6 (3)	O2—C26—C25	115.6 (4)
N1—C11—C10	128.3 (3)	C17—C26—C25	119.7 (3)
N1—C11—H11A	115.8	C27—O3—H1	111.3
C10—C11—H11A	115.8	O3—C27—C28	114.8 (5)
N1—C12—C13	114.3 (4)	O3—C27—H27A	108.6
N1—C12—H12A	108.7	C28—C27—H27A	108.6
C13—C12—H12A	108.7	O3—C27—H27B	108.6
N1—C12—H12B	108.7	C28—C27—H27B	108.6
C13—C12—H12B	108.7	H27A—C27—H27B	107.6
H12A—C12—H12B	107.6	C27—C28—H28A	109.5
C12—C13—C14	115.9 (4)	C27—C28—H28B	109.5
C12—C13—H13A	108.3	H28A—C28—H28B	109.5
C14—C13—H13A	108.3	C27—C28—H28C	109.5
C12—C13—H13B	108.3	H28A—C28—H28C	109.5
C14—C13—H13B	108.3	H28B—C28—H28C	109.5
H13A—C13—H13B	107.4		
			
O2—Cu1—O1—C1	160.0 (3)	C4—C9—C10—C11	179.8 (4)
N1—Cu1—O1—C1	9.4 (3)	C12—N1—C11—C10	−178.0 (4)
N2—Cu1—O1—C1	−108.7 (4)	Cu1—N1—C11—C10	−5.7 (6)
O1—Cu1—O2—C26	153.9 (3)	C1—C10—C11—N1	7.9 (7)
N1—Cu1—O2—C26	−117.1 (3)	C9—C10—C11—N1	−173.2 (4)
N2—Cu1—O2—C26	6.0 (3)	C11—N1—C12—C13	−101.9 (4)
O2—Cu1—N1—C11	−89.3 (4)	Cu1—N1—C12—C13	85.4 (4)
O1—Cu1—N1—C11	−1.9 (3)	N1—C12—C13—C14	−81.5 (5)
N2—Cu1—N1—C11	149.5 (3)	C12—C13—C14—C15	69.1 (5)
O2—Cu1—N1—C12	82.7 (4)	C16—N2—C15—C14	−102.7 (4)
O1—Cu1—N1—C12	170.2 (3)	Cu1—N2—C15—C14	83.6 (4)
N2—Cu1—N1—C12	−38.4 (3)	C13—C14—C15—N2	−83.3 (5)
O2—Cu1—N2—C16	−0.6 (3)	C15—N2—C16—C17	−178.3 (3)
O1—Cu1—N2—C16	−89.9 (4)	Cu1—N2—C16—C17	−4.8 (5)
N1—Cu1—N2—C16	154.5 (3)	N2—C16—C17—C26	6.2 (6)
O2—Cu1—N2—C15	172.5 (3)	N2—C16—C17—C18	−175.4 (3)
O1—Cu1—N2—C15	83.3 (3)	C26—C17—C18—C19	178.1 (3)
N1—Cu1—N2—C15	−32.3 (3)	C16—C17—C18—C19	−0.3 (5)
Cu1—O1—C1—C10	−9.7 (6)	C26—C17—C18—C23	−1.3 (5)
Cu1—O1—C1—C2	170.9 (3)	C16—C17—C18—C23	−179.7 (3)
O1—C1—C2—C3	−179.8 (4)	C23—C18—C19—C20	−1.1 (6)
C10—C1—C2—C3	0.8 (6)	C17—C18—C19—C20	179.4 (4)
C1—C2—C3—C4	−1.9 (7)	C18—C19—C20—C21	1.0 (7)
C2—C3—C4—C9	1.4 (7)	C19—C20—C21—C22	−0.9 (8)
C2—C3—C4—C5	−177.8 (4)	C20—C21—C22—C23	1.0 (8)
C3—C4—C5—C6	178.7 (4)	C19—C18—C23—C24	−178.4 (3)
C9—C4—C5—C6	−0.5 (7)	C17—C18—C23—C24	1.1 (5)
C4—C5—C6—C7	0.0 (8)	C19—C18—C23—C22	1.2 (5)
C5—C6—C7—C8	1.0 (8)	C17—C18—C23—C22	−179.4 (4)
C6—C7—C8—C9	−1.4 (8)	C21—C22—C23—C18	−1.1 (7)
C7—C8—C9—C4	0.8 (7)	C21—C22—C23—C24	178.4 (5)
C7—C8—C9—C10	−178.4 (4)	C18—C23—C24—C25	0.0 (6)
C3—C4—C9—C8	−179.1 (4)	C22—C23—C24—C25	−179.5 (4)
C5—C4—C9—C8	0.1 (6)	C23—C24—C25—C26	−1.0 (6)
C3—C4—C9—C10	0.2 (6)	Cu1—O2—C26—C17	−6.3 (5)
C5—C4—C9—C10	179.4 (4)	Cu1—O2—C26—C25	174.7 (3)
O1—C1—C10—C11	0.3 (6)	C16—C17—C26—O2	−0.3 (6)
C2—C1—C10—C11	179.7 (4)	C18—C17—C26—O2	−178.6 (3)
O1—C1—C10—C9	−178.6 (4)	C16—C17—C26—C25	178.7 (3)
C2—C1—C10—C9	0.8 (6)	C18—C17—C26—C25	0.4 (5)
C8—C9—C10—C1	178.0 (4)	C24—C25—C26—O2	179.8 (4)
C4—C9—C10—C1	−1.2 (6)	C24—C25—C26—C17	0.7 (6)
C8—C9—C10—C11	−1.0 (6)		

### Hydrogen-bond geometry (Å, °)

**Table:**

*D*—H···*A*	*D*—H	H···*A*	*D*···*A*	*D*—H···*A*
O3—H1···O1	0.90	1.95	2.837 (4)	167
C12—H12A···O2^i^	0.97	2.52	3.395 (5)	150

Symmetry codes: (i) −*x*+2, *y*, −*z*+1/2.
